# On the Importance of Recognizing the Conditions Known as Sthenic and Asthenic, Etc.

**Published:** 1883-07-20

**Authors:** Harvey L. Byrd

**Affiliations:** Baltimore


### ON THE IMPORTANCE OF RECOGNIZING THE CONDITIONS KNOWN AS STHENIC AND ASTHENIC, IN DIFFERENTIATING THE CAUCASIAN AND AFRICAN RACES; AND IN DISEASES GENERALLY.

BY HARVEY L. BYRD, M.D., BALTIMORE.

There are two good old words with explicit signification, which were much and properly used for the most part by our grandfathers in the profession, that seem to have lost their value in a great measure in the estimation of some of our modern teachers and writers upon medical subjects, which might be profitably restored to their former prominence and importance in our professional literature of the present day. Should this brief communication, therefore, produce no other beneficial result than that of recalling attention to their significance and their peculiar fitness to occupy an enduring place in our nomenclature, and thus to keep the two widely separated and almost diametrically opposite states or conditions of the human organism in health and in disease, always prominently and perspicuously before the medical mind, a most important step will have been taken toward wiping away some of the flimsy drapery with which certain morbid conditions have been sought to be clothed by a few modern writers, and many of the hair-breadth distinctions between diseases, attempted to be made by others, bridged over or annihilated altogether. The import of the good old Greek, *sthenos* and *asthenos*, stand out in bold relief before the mind of the medical philosopher, of much practical experience in the profession, whenever he is called to the bedside of disease. And like the Pillars of Hercules, they must ever mark the entrance into the great sea of successful professional practice, where their significance is obeyed as it should be. It is not, therefore, claiming too much for those words to add, that a correct knowledge of the conditions which they represent, establishes the only true basis or foundation, upon which an accurate diagnosis can be formed, and consequently a correct therapeusis instituted in the treatment of diseases.

The value and importance, then, for the revival and a more general use of those terms in our discussions and treatment of the thousand ills to which flesh is heir, must be obvious to the intelligent mind upon a moment's consideration; particularly as they may be readily observed as intimately associated with the action of morbific agents in the development of disease, and sometimes, if not frequently, important factors as predisposing, and, possibly, even the existing cause of certain diseases in either race, and among many conditions of mankind. But there is absolute necessity for the use of these words, if we would appreciate at their proper value certain normal or natural states and conditions as we find them existing in the *primordial* types of the genus homo. And long experience has satisfied my mind completely that professional success of a high order is unattainable where the Caucasian and African races are contemporaneously attended either in hospital or private practice, without heeding the natural tendency of disease to a sthenic state in the white and an asthenic condition in the negro race. This state of things is conspicuously marked in endemic and epidemic visitations, when the two races are under the action of the same morbific agent or influence for a certain space of time. The natural and radically distinct differences and peculiarities, anatomical and physiological, which exist between the negro and the white man, have been already sufficiently referred to in a previous article in this journal, when speaking of the primordial races of man; and hence it will only be necessary to say a few words, a little further on in this paper, on the effect of *materies morbi*, and the action of therapeutic agents upon the respective races to render the entire matter clearly intelligible to the reader.

As both the white man and negro are exotic, and have resided contemporaneously in this country for a sufficiently long period of time under the same and similar climatic, hygienic and physical surroundings to be equally well known, they are, therefore, in a suitable condition to be studied in regard to their predisposition to, and their ability to withstand, the influence of morbific agents, when acting as chief factors in the development of endemic and epidemic diseases.

Whilst presenting some leading facts in support of the foregoing premises, I shall take occasion to introduce the action of therapeutic agents, as being fully in harmony with the laws which establish and control diversity in the races to which they apply; and thus develop the utility, not to say imperative necessity, for carefully regarding the sthenic and asthenic conditions as factors of the highest value in the diseases to which the races are liable. And, therefore, the absolute demand for a modification of treatment in the Caucasian and African races when suffering from the same disease.

To what has been said already of the status of the two races must now be added the distinctions of age and sex, in order that the parallelism may be rendered as complete as possible, and thus the differentiations be perspicuously brought forth and made of practical advantage to the profession in a clearer presentation of the value of symptoms and signs at the bedside. As already intimated, the white and black races are not equally liable to impressions from morbific agents and influences.

Thus, to *Cholera*—For twenty or more years preceding the late civil war, cholera occurred from time to time, endemically, among the negroes on the rice plantations of South Carolina and Georgia, assuming even epidemic proportions as it extended to adjacent or contiguous plantations; and though the overseer and his subordinates and their families even, remained upon the plantations continuously during such visitations, they enjoyed almost complete immunity from the disease. The negroes all the while manifesting the chain of phenomena so characteristic of Asiatic cholera in a malignant form.

*Second.*—YELLOW FEVER.—As that disease prevailed epidemically in the cities of the South, in ante-bellum times, the white or Caucasian race, was the material upon which it seemed pleased to feed ; and the fairer the type, all other things being equal, the greater the fatality of the disease ; while the African, or negro race, was nearly exempt from its ravages. In my own experience, in the treatment of hundreds of yellow fever patients, I never saw a case of black vomit in a negro.

*Third.*—MALARIA.—While he was liable to be attacked with remittent and intermittent fever, the negro was very far less exposed to the effects of malaria than the white man, under the same circumstances.

*Fourth.*—INFLAMMATORY DISEASES.——The negro suffered much less frequently from inflammation of the serous and fibrous tissues than the white race, and much less also from troubles of that kind in the mucous surfaces ; and though attacked with pneumonia to a considerable extent, when it appeared to prevail epidemically in the South, he did not suffer in the same ratio with the white man, though far more exposed to inclement weather and atmospheric vicissitudes. The parallelism and differentiations of the disease to which the two races are exposed might be extended almost indefinitely ; but it is thought that a sufficient number of facts have been adduced to satisfy the wishes of any who might be pursuing this branch of professional inquiry, and I will therefore hasten on, as fast as practicable, to the other salient points adverted to in the earlier portion of this paper.

Any, and as far as I know, every disease that affects the white and negro races, in common, assumes a lower grade of action much sooner, under the same circumstances, in the latter than in the former race, when that state is not conspicuously obvious *ab initio.*

Again, I feel quite sure that I can state, with great accuracy and propriety of language, that the converse of the foregoing observations is true of diseases generally in the white race—certainly so when contrasted with morbid conditions as found in the former race. Hence it would seem to be logically certain, that while a disease might exhibit a higher or lower grade of action, in either the Caucasian or the African, under certain circumstances, and at different times, the law of depending upon difference in race is, that the type of disease in the white race, is sthenic, and it is asthenic in the negro, under the same, or as nearly as possible the same, circumstances. These types will of course suffer modification or exaltation, and become more or less sthenic or asthenic, under different or varying influences ; all of which are, however, too well known or easily recognized by the profession generaly to require that more should be said in that connection. But these facts do not lessen the value of the preceding statements in any way, regarding the tendency of all diseases to assume a sthenic or an asthenic type, whether they occur in the white or negro races.

The constancy of the action of this law was strikingly exemplified in my experience in the South during the civil war, both in regard to the symptoms of disease and the treatment necessary for the two races, under as nearly the same circumstances as it is possible for them to appear. A proper appreciation of the foregoing facts is necessary in order that therapeutic applications may be judiciously and properly made in the treatment of diseases. In fact, it may be safely said that this most important consummation cannot be certainly reached in any other way. To illustrate its value, I will state a few facts that have been repeatedly observed in my professional experience. Thus, sedatives and depressants and antiphlogistics generally, whilst often called for in the treatment of acute and recent cases of disease, and manifest such prompt and decisive benefit when applied in the Caucasian, as a rule produce no good results in the negro, unless they should be used with great caution, and then suspended promptly upon the slightest evidence of depression taking place; and it may be safely said that such remedies are often productive of positive harm in his case. This fact is particularly and strikingly exemplified in the effects of bloodletting and in the action of veratrum viride—two of the most potent sedatives and at the same time valuable agents where their action is called for in the white race, are almost always attended with harm in the negro, and especially so when carried to the extent found useful and necessary in the Caucasian. Thus, no agents act more satisfactorily or philosophically in the subduction of acute inflammatory symptoms in the white race, or are more urgently called for in the scientific treatment of such cases, than bloodletting and arterial sedatives; and yet, if they are resorted to for a similar purpose in the negro, the greatest caution and circumspection are imperatively demanded in order that harm shall not result from their use. In the rare cases in the negro which seem to warrant active antiphlogistic treatment, tonics and stimulants are required at an early period in their management. Opium and its preparations are badly borne by the African, as a rule, and the same is true, in an equal degree, of the entire narcotic class of remedial agents in their action upon his organism.

This is an exceedingly interesting branch of professional inquiry, and though the field is so inviting as to tempt me to detain the reader much further in the presentation of many other valuable facts gleaned in it during more than two decades of active practice upon and near the rice plantations of South Carolina and Georgia, where, I may add, that in ante-bellum days the material and the opportunities for turning it to practical and valuable account were of the most abundant character, yet it seems necessary that, after a few more words or statements of closely allied facts, this hastily written article should be brought to a close.

The hybrid—mulatto—is a factor of considerable importance to physicians who encounter him in practice to much extent. But when he is considered as partaking of the structure and *nature* of his progenitors in a nearly equal degree, it will be readily perceived that he occupies a place intermediate, or between the Caucasian and African races, in other respects also. His immunity and his susceptibility to the diseases of his progenitors, and his impressibility to the action of therapeutic agents, is also of an intermediate grade between that of the races from which he sprang.—*N. Y. Med. Record.*

